# The Biological Function, Mechanism, and Clinical Significance of m6A RNA Modifications in Head and Neck Carcinoma: A Systematic Review

**DOI:** 10.3389/fcell.2021.683254

**Published:** 2021-05-31

**Authors:** Feng-Yang Jing, Li-Ming Zhou, Yu-Jie Ning, Xiao-Juan Wang, You-Ming Zhu

**Affiliations:** Key Laboratory of Oral Diseases Research of Anhui Province, Department of Dental Implant Center, Stomatologic Hospital & College, Anhui Medical University, Hefei, China

**Keywords:** head and neck squamous cell carcinoma, RNA methylation, N6-methyladenosine, epigenetics, tumor microenvironment, targeted therapy

## Abstract

Head and neck squamous cell carcinoma (HNSCC) is one of the most common cancers, yet the molecular mechanisms underlying its onset and development have not yet been fully elucidated. Indeed, an in-depth understanding of the potential molecular mechanisms underlying HNSCC oncogenesis may aid the development of better treatment strategies. Recent epigenetic studies have revealed that the m6A RNA modification plays important roles in HNSCC. In this review, we summarize the role of m6A modification in various types of HNSCC, including thyroid, nasopharyngeal, hypopharyngeal squamous cell, and oral carcinoma. In addition, we discuss the regulatory roles of m6A in immune cells within the tumor microenvironment, as well as the potential molecular mechanisms. Finally, we review the development of potential targets for treating cancer based on the regulatory functions of m6A, with an aim to improving targeted therapies for HNSCC. Together, this review highlights the important roles that m6A modification plays in RNA synthesis, transport, and translation, and demonstrates that the regulation of m6A-related proteins can indirectly affect mRNA and ncRNA function, thus providing a novel strategy for reengineering intrinsic cell activity and developing simpler interventions to treat HNSCC.

## Introduction

Head and neck squamous cell carcinoma (HNSCC) is the sixth most common cancer worldwide, with an incidence of approximately 700,000 confirmed cases annually (Ferris et al., 2018). Head and neck tumors comprise neoplasms of the neck; ear, nose, and throat; and oral and maxillofacial regions. While thyroid carcinoma is the most common endocrine malignancy, nasopharyngeal carcinoma and hypopharyngeal squamous cell carcinoma are common in otorhinolaryngological practice and oral carcinomas, such as tongue, gingival, and buccal cancer are the most common malignant neoplasms of the head and neck region. Although standard treatment strategies, such as surgery, chemotherapy, and radiotherapy have considerably improved the prognosis of patients with HNSCC, the rate of overall survival remains low (Wood et al., 2017). Recent advancements in molecular biology have allowed researchers to study the molecular mechanisms underlying tumorigenesis in greater detail. Consequently, targeted therapies based on the regulation of these molecular mechanisms have received increasing interest for treating cancer (Biktasova et al., 2017).

Over 100 types of post-transcriptional modification have been identified in eukaryotic cells (He, 2010; Cantara et al., 2011; Globisch et al., 2011), among which N6-methyladenosine (m6A), which was discovered in the 1970s, is the most common (Roundtree et al., 2017). m6A RNA methylation accounts for approximately 50% of all methylated nucleotides and occurs in 0.1–0.4% of RNA sequences in proximity to the stop codon in the 3’-untranslated region (3’-UTR) and in large exons (Desrosiers et al., 1974; Wei et al., 1975). Although the multi-component methyltransferase complex that catalyzes m6A formation was first reported in 1994 (Bokar et al., 1994), the biological function of m6A varies according to the environment and its precise mechanism of action remains poorly understood.

In this review, we briefly describe the mechanisms underlying m6A modification, as well as its important roles in RNA synthesis, transport, and translation. Next, we summarize the role of m6A modification in various types of HNSCC as well as its regulatory roles in immune cells within the tumor microenvironment. Finally, we review the development of potential therapeutic targets based on the regulatory functions of m6A that could improve the treatment of HNSCC.

## m6A RNA Modification

**FIGURE 1:**
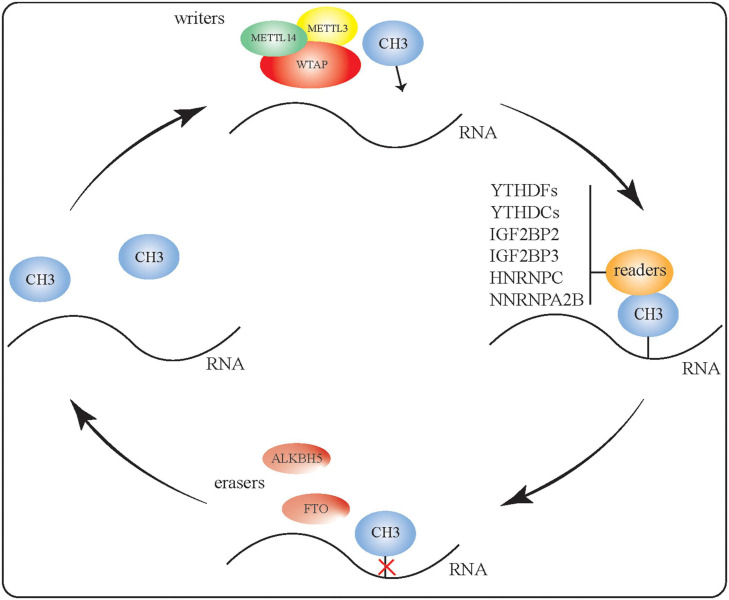
RNA m6A modification. Methyl groups are added, recognized, and removed by writer, reader, and eraser proteins, respectively. The methyltransferases METTL3, METTL14, and WTAP can form complexes to regulate RNA methylation. Binding proteins such as YTH domain proteins and HNRNP proteins can identify and bind to methylated RNA. The two m6A eraser proteins—FTO and ALKBH5—perform RNA demethylation, making the m6A RNA modification a reversible and dynamic process.

Methyl groups are added, recognized, and removed by writer, reader, and eraser proteins, respectively, to generate m6A-modified RNA (Figure 1). To regulate RNA methylation, the methyltransferases METTL3, METTL14, and WTAP can form complexes with various binding proteins, such as YT521-B homologous (YTH) domain proteins and heterogeneous nuclear ribonucleoprotein (HNRNP) family proteins, which can identify and bind to methylated RNA. Conversely, m6A eraser proteins can catalyze RNA demethylation as part of the reversible dynamic process of m6A modification. Here, we briefly summarize our current understanding of the writer, reader, and eraser proteins that contribute toward m6A modification.

### m6A Writers

Multiple cellular methyltransferases are known to mediate m6A modification, as listed in Table 1. In particular, METTL3 and METTL14 can form a stable complex with WTAP at the methylation site in mammalian cells (Liu et al., 2014). METTL14, a pseudo methyltransferase (Ondo et al., 2020) that lacks enzymatic activity, can stabilize METTL3 and recognize target RNA (Śledź and Jinek, 2016; Wang et al., 2016a, b), thereby acting as a connector between the RNA substrate and METTL3. However, the precise mechanism underlying the action of methyltransferases remains unclear and further studies are required to provide a more comprehensive understanding.

**TABLE 1:** Enzymes mediating N6-methyladenosine (m6A) deposition.

**Symbol**	**Full name**	**HGHG ID**	**Function**	**Location**
METTL3	Methyltransferase-like 3	17563	Writer	14q11.2
METTL14	Methyltransferase-like 14	29330	Writer	4q26
METTL16	Methyltransferase-like 16	29330	Writer	17p13.3
WTAP	WT1-associated protein	16846	Writer	6q25.3
ZC3H13	Zinc finger CCCH-type containing 13	20368	Writer	13q14.13
ZCCHC4	Zinc finger CCHC-type containing 4	22917	Writer	4p15.2
RBM15	RNA-binding motif protein 15	14959	Writer	1p13.3
RBM15B	RNA-binding motif protein 15B	24303	Writer	3p21.2
YTHDF1	YTH N6-methyladenosine RNA-binding protein 1	15867	Reader	20q13.33
YTHDF2	YTH N6-methyladenosine RNA-binding protein 2	31675	Reader	1p35.3
YTHDF3	YTH N6-methyladenosine RNA-binding protein 3	26465	Reader	8q12.3
YTHDC1	YTH domain-containing 1	30626	Reader	4q13.2
YTHDC2	YTH domain-containing 2	24721	Reader	5q22.2
IGF2BP2	Insulin-like growth factor 2 mRNA-binding protein 2	28867	Reader	3q27.2
IGF2BP3	Insulin-like growth factor 2 mRNA-binding protein 3	28868	Reader	7p15.3
HNRNPC	Heterogeneous nuclear ribonucleoprotein C	5035	Reader	14q11.2
HNRNPA2B1	Heterogeneous nuclear ribonucleoprotein A2/B1	5033	Reader	7p15.2
FTO	FTO alpha-ketoglutarate-dependent dioxygenase	24678	Eraser	16q12.2
ALKBH5	alkB homolog 5, RNA demethylase	25996	Eraser	17p11.2

*HGHG, HUGO gene nomenclature committee.*

### m6A Readers

The most important function of m6A is the recruitment of m6A-binding proteins to RNA. Recent studies have shown that m6A can be recognized by YT521B homology domain proteins and eukaryotic initiation factor 3 (eIF3) (Jiang et al., 2021). The mammalian genome contains five YTH domain proteins: YTHDC1 (DC1), YTHDC2 (DC2), YTHDF1 (DF1), YTHDF2 (DF2), and YTHDF3 (DF3). YTHDF2 is the most abundant DF family protein in almost all cell types and was the first m6A reader protein reported to facilitate the localization of mRNA to RNA decay sites (Wang et al., 2014a). Conversely, YTHDF1-mediated promoters are known to improve translation efficiency, whereas YTHDF3 plays an important role during the initial stages of translation; however, the detailed mechanism remains unclear (Li et al., 2017a). Various other binding proteins, such as hnRNPA2B1 and hnRNPC, have also been recently identified (Table 1) and their functions are described in detail throughout this review.

### m6A Erasers

Currently, FTO (alpha-ketoglutarate dependent dioxygenase), and ALKBH5 (alkB homolog 5) are the only known m6A eraser proteins. FTO is an m6A demethylase that has previously been associated with obesity in humans (Jia et al., 2011). Indeed, this study demonstrated that the m6A modification is reversible and dynamic, not just formation-related, and thus greatly increased research on RNA methylation. The FTO homolog, ALKBH5 is also a m6A demethylase (Rubio et al., 2018; Table 1); however, limited evidence is available regarding its role in the direct demethylation of specific m6A loci in mRNA (Meyer and Jaffrey, 2017). Although m6A demethylases have been recognized to play an important role in regulating the extent of m6A RNA modification, our current understanding of their precise activity is limited and further studies are required to identify their other physiological roles.

Despite the limitations in our current understanding of the precise mechanisms underlying m6A RNA modification, various studies have examined the effects of m6A modification on the regulation of RNA transcripts.

## Roles of m6A in RNA Regulation

**FIGURE 2:**
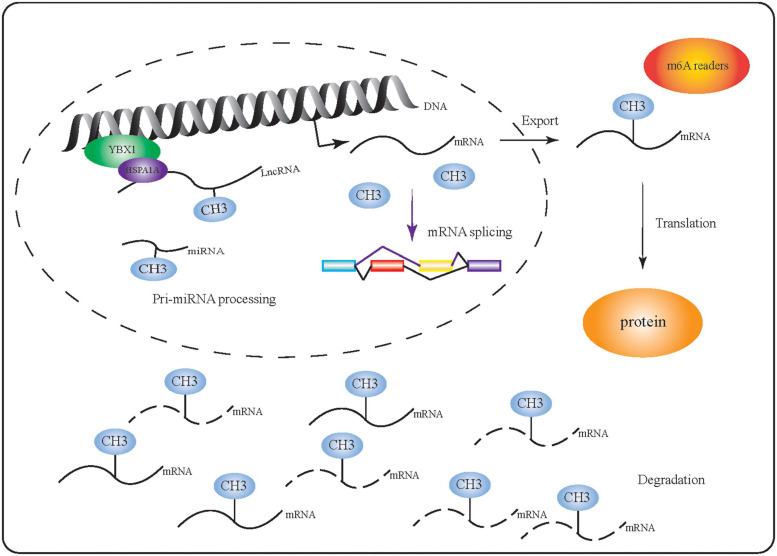
m6A-modified RNA performs multiple functions. m6A-related proteins (including writers, readers, and erasers) affect mRNA function by regulating RNA synthesis, splicing, export, translation, degradation, and miRNA maturation.

m6A is thought to be the most abundant chemical modification of mammalian mRNA and non-coding RNA and is involved in the regulation of several physiological and disease processes (Perry et al., 1975; Fomenkov et al., 2020; Kontur et al., 2020; Wang and Lu, 2021). In particular, m6A can facilitate or be directly involved in RNA processing (Figure 2). Comprehensive methods to identify transcriptome-wide m6A profiles have improved our understanding of this modification and have shown that m6A can selectively regulate transcription. Proteins such as HnRNP, which can selectively bind to m6A-modified mRNAs in response to physiological factors (Liu et al., 2015; Jin et al., 2021), regulate mRNA function by modulating RNA splicing, export, translation, degradation, as well as miRNA maturation (Huang et al., 2018; Zhang et al., 2020; Chen et al., 2021a; Jin et al., 2021; Song et al., 2021). Here, we examine the effects of m6A on the function of RNAs throughout different stages of their processing.

### m6A in RNA Splicing

Previous studies have shown that m6A-modifications can spatially overlap with the splicing enhancer region and act as a pre-RNA splicing regulator to promote alternative mRNA splicing (Zhao et al., 2014). In addition, METTL16 was found to promote RNA splicing by targeting the downstream mRNAs and non-coding RNAs (Warda et al., 2017). During splicing, METTL16 can induce m6A modification in the 3′-UTR of mRNA and A43 modification in U6 nuclear RNA with 50 base pairs of pre-mRNA, indicating that METTL16 plays important roles in determining mRNA stability and splicing (Aoyama et al., 2020; Ovcharenko et al., 2021). The inhibition of other m6A methyltransferases was also shown to affect gene expression and alternative splicing patterns (Dominissini et al., 2012), while METTL3 downregulation alters RNA splicing in pancreatic cancer (Taketo et al., 2018). Furthermore, YTHDC1 has been shown to recruit the splicing factor SRSF3 to m6A-modified pre-RNAs to promote splicing (Kasowitz et al., 2018), suggesting that m6A binding proteins can also regulate RNA splicing.

In addition, demethylases have been reported to play important roles in regulating m6A-mediated RNA splicing. For instance, pre-mRNA in the preferential binding intron region of FTO is located close to the alternative splicing exon and polyA site (Hess et al., 2013), while the alternative splicing of nuclear pre-mRNA can be regulated by SRSF2 binding (Bartosovic et al., 2017). The removal of m6A by FTO inhibits SRSF2 recruitment and promotes exon 6 skipping, resulting in a short subtype of Runt-related transcription factor 1 (Ben-Haim et al., 2015). The downregulation of ALKBH5 can also enhance exon jumping and induce the rapid degradation of abnormally spliced transcripts (Tang et al., 2018). Furthermore, ALKBH5 can affect the phosphorylation of alternative splicing factor/splicing factor 2 (ASF/SF2) in the nucleus, whose hyperphosphorylated form is involved in pre-mRNA splicing (Zheng et al., 2013).

### m6A in RNA Nuclear Export

Recent studies have shown that m6A writer, reader, and eraser proteins can promote RNA nuclear export. In particular, METTL3, METTL14, and WTAP-mediated m6A modification were found to promote the nuclear-to-cytoplasmic translocation of mRNA (Fustin et al., 2013). Meanwhile, the m6A reader protein YTHDC1 can promote the binding of RNA to SRSF3 and the mRNA export receptor Nxf1, thereby targeting m6A-modified mRNA for nuclear export (Wang et al., 2015). Similarly, ALKBH5 can promote mRNA export by reducing the phosphorylation of ASF/SF2, enabling it to bind to the TAP-p15 complex, a general mRNA nuclear export receptor that binds to and translocates mRNA (Li and Manley, 2005; Michlewski et al., 2008; Zheng et al., 2013).

### m6A in mRNA Translation

m6A modification in the 5′- or 3′-UTR of mRNAs and non-coding RNAs has been found to regulate gene expression (Selmi et al., 2021; Yao et al., 2021) and promote the translation of 5’-UTR-methylated mRNAs (Shen et al., 2020; Zhang et al., 2021). Moreover, translational regulation by m6A has been reported to play a key role in several cancers. In acute myeloid leukemia (AML), m6A upregulates the expression of the MYB proto-oncogene, MYC proto-oncogene, BCL2, phosphatase and tensin homolog, and SP1 transcription factor, which in turn enhance the binding ability and translation efficiency of the oncogenic RNA and ribosome to facilitate tumorigenesis (Li et al., 2017b; Wang et al., 2020a; Qing et al., 2021; Yankova et al., 2021). The upregulation of the m6A reader protein METTL3 has also been shown to enhance mRNA translation (Schumann et al., 2016; Coots et al., 2017), whereas its downregulation selectively inhibits mRNA translation (Coots et al., 2017), decreases AF4/FMR2 family member 4 and MYC proto-oncogene translation in bladder cancer (Cheng et al., 2019), and increases zinc finger protein 750 and fibroblast growth factor 14 translation in nasopharyngeal carcinoma (Zhang et al., 2018). Furthermore, METTL3 was found to increase m6A deposition in its target genes in the human AML cell line MOLM-13, as well as myelomatosis and B-cell lymphoma 2 (Vu et al., 2017), and has been reported to enhance the translation of its target mRNA, *tafazzin*, independently of its methyltransferase activity in lung cancer cells (Lin et al., 2016).

m6A-induced translational regulation also plays a key role in normal physiological function. For instance, m6A has been observed in the transcripts of key regulatory factors, such as ZBTB16, ID4, Dnmt3b, and Sohlh2, in spermatogonial stem cells/progenitor cells and regulates their transcription and translation to coordinate protein synthesis. Thus, the m6A modification is essential for spermatogenesis in mammals (Lin et al., 2017). Studies have shown that human YTHDF1 selectively recognizes m6A-modified mRNAs and enhances translation by interacting with initiation factors and ribosomes, indicating that YTHDF1 directly promotes translation initiation and transports cellular mRNAs to the translation machinery (Tassinari et al., 2021). During mRNA translation, the recognition and binding of m6A by YTHDFs also results in enhanced protein synthesis (Shi et al., 2017; Su et al., 2021). In particular, YTHDC2 selectively binds to m6A using its consensus motif to improve the translation efficiency and reduce the abundance of the target mRNA (Hsu et al., 2017; Wojtas et al., 2017).

Heat shock proteins are also involved in m6A-mediated translation regulation. Under heat shock conditions, m6A preferentially binds to the 5’-UTR of stress-induced transcripts, such as heat shock protein family H (Hsp110) member 1. Moreover, increased 5′-UTR m6A modification can further enhance the initiation of cap-independent translation (Zhou et al., 2015). m6A modification can also promote translational priming by interacting with the initiators eIF3, CBP80, and eIF4E in an RNA-independent manner. Cellular stress increases the 5′-UTR m6A modification of RNA transcripts, which in turn promotes mRNA translation by directly binding to eIF3 in a YTHDF1-independent manner (Liu et al., 2020a). Translation initiation typically requires the eIF4 protein complex, particularly the cap-binding protein eIF4e (Jackson et al., 2010). However, m6A-modified mRNA can recruit eIF3 in the absence of eIF4E and other components of the eIF4 complex, allowing the m6A-modified mRNAs to be translated in the presence of eIF3 and other priming factors (Orouji et al., 2020). Subsequent studies have also confirmed that m6A is required to recruit eIF3 to mRNA during protein translation (Hwang et al., 2021).

### m6A in mRNA Degradation

The degree of m6A modification can determine the stability of cytoplasmic mRNA (Ke et al., 2017), with METTL3-, METTL14-, and WTAP-mediated m6A modification all reducing mRNA stability (Schwartz et al., 2014; Geula et al., 2015; Knuckles et al., 2017; Bertero et al., 2018). For instance, METTL3 or METTL14 downregulation in T cells inhibits the m6A mRNA modification and increases the expression of suppressor of cytokine signaling (SOCS) family mRNAs (Jiang et al., 2020).

YTHDF family proteins can also accelerate the decay of m6A-modified mRNA transcripts (Liu et al., 2020b) or target mRNAs (Wang and He, 2014b). For example, YTHDC1 recognizes the m6A-modified 3’-UTR of MAT2A to induce methyl donor S-adenosyl methionine-mediated MAT2A mRNA degradation (Shima et al., 2017). YTHDF2 can also recognize m6A-modified SOCS2 and accelerate its degradation to induce tumorigenesis (Chen et al., 2018); however, the downregulation of m6A methyltransferase weakens the interaction between YTHDF2 and the target mRNA, increasing SOCS2 mRNA stability (Mapperley et al., 2021). However, studies using YTHDF1-knockout cells have shown that YTHDF1 has minimal effect on mRNA stability (Wang et al., 2015). One study found that all YTHDF proteins can increase mRNA stability and protein expression (Kennedy et al., 2016), yet experiments conducted by other researchers showed that all three YTHDF proteins can initiate mRNA degradation and dealkylation (Du et al., 2016). These varying results have raised questions regarding the functions of YTHDF proteins. Meanwhile, a study on the m6A reader protein IGF2BP1/2/3 found that it recognized the common GG (m6A) C sequence via its K homologous domain and enhanced the stability and translation of its target mRNA in an m6A-dependent manner under normal and stress conditions (Huang et al., 2018).

The m6A eraser protein, FTO, was found to increase the stability of MYC mRNA by inhibiting YTHDF2-mediated RNA decay (Su et al., 2018; Weng et al., 2018), while ALKBH5 is also known to regulate mRNA stability. In particular, ALKBH5 has been found to localize at nuclear sites, regulate the assembly/modification of mRNA processing factors, demethylate m6A mRNA, and regulate mRNA export and stability (Zheng et al., 2013). In ALKBH5-deficient spermatocytes, increased nuclear RNA efflux significantly increases cytoplasmic RNA levels as well as the synthesis of newborn RNA, while decreasing overall RNA stability, leading to spermatocyte apoptosis (Zheng et al., 2017).

Functional proteins such as transcription factors can also affect mRNA stability by regulating the degree of m6A modification in mRNA transcripts. For example, zinc finger protein 217 (ZFP217) activates the transcription of key pluripotent genes and regulates the m6A modification of the corresponding transcripts. Thus, ZFP217 depletion enhances the m6A modification in *Nanog*, *Sox2*, *Klf4*, and *c-Myc* mRNAs to accelerate their degradation, thereby disrupting the self-renewal of embryonic stem cells and somatic reprogramming (Aguilo et al., 2015).

METTL3 is also known to methylate pri-miRNAs, tag them for identification and processing, and promote global miRNA maturation in a cell type-independent manner (Bhat et al., 2020; Yi et al., 2020a). In breast cancer, METTL3 recognizes pri-miRNAs via the microprocessor protein DGCR8, increases mature miRNA levels, and decreases untreated pri-miRNA levels (Zhong et al., 2020). Other methylases are also involved in miRNA regulation. For instance, the activation of protease activated receptor 2 decreases miR-125b levels via NOP2/Sun RNA methyltransferase family member 2 (NSUN2), which methylates the miR-125b precursor, interferes with its processing, and reduces mature miR-125b levels (Yuan et al., 2014). m6A modification can also be performed by identifying DGCR8 and labeling the original RNA in a METTL3/m6A-dependent manner. In particular, the m6A binding protein hnRNPA2B1 can recruit DGCR8 to RNA by targeting the m6A site, thus playing an important role in promoting pri-miRNA processing (Alarcón et al., 2015; Chen et al., 2020); however, the precise mechanism remains poorly understood. Future studies are therefore required to determine whether m6A modification plays a key role in miRNA maturation and whether miRNA maturation could be regulated by targeting the m6A modification sites.

### Other Functions of m6A in RNA Regulation

In addition to its roles in the processing of RNAs, the m6A modification of mRNAs and ncRNAs also plays key roles in the growth and development of various tumors. For instance, HBXIP upregulates METTL3 expression in breast cancer cells by inhibiting miRNA-let-7g, which downregulates METTL3 expression by targeting its 3’-UTR. METTL3 subsequently increases *HBXIP* expression by increasing m6A modification, thereby creating a positive feedback loop consisting of HBXIP/miR-let-7g/METLL3/HBXIP that accelerates breast cancer cell proliferation (Cai et al., 2018). FTO also interacts with METTL3 to regulate polyA site and 3’-UTR lengths (Begik et al., 2020), which can also be altered by YTHDC1 knockout (Chen et al., 2021b). Thus, all of these factors may contribute toward tumor development. Indeed, AML can be initiated by the chromosomal translocation of RNA binding motif protein 15 (RBM15), another component of the m6A writer complex (also known as OTT1), with myelin (Mercher et al., 2001). In hepatocellular carcinoma, reduced miRNA-126 m6A modification affects its function as a ceRNA (competing endogenous RNAs) and decreases its binding capacity, thus promoting tumor development (Ma et al., 2017).

Together, these studies demonstrate that m6A modification plays an important role in RNA synthesis, transport, and translation, and that the regulation of m6A-related proteins can indirectly affect mRNA and ncRNA function. Moreover, m6A modification also plays an important role in the occurrence and development of various tumors by regulating miRNA processing, mRNA translation, and RNA stability. These findings suggest a novel strategy for reengineering intrinsic cell activity and simplifying intervention measures that could provide novel strategies for the diagnosis and treatment of cancer. However, our understanding of the roles and mechanisms of m6A modification is currently very limited and further studies are required to elucidate these aspects in more detail.

## Roles of m6A Modification in HNSCC

Although current evidence suggests that m6A plays important roles in cancer, to our knowledge, no systematic reviews have yet described the role of m6A modification in HNSCC. Herein, we discuss the mechanism of m6A in several common HNSCCs and aim to provide novel ideas for future research regarding the pathogenesis and development of HNSCC (Table 2).

**TABLE 2:** Regulation of m6A modification in head and neck squamous cell carcinoma (HNSC).

**Cancer**	**m6A regulators**	**Regulation**	**Mechanism**	**References**
THCA	METTL3	Up	METTL3 can activate the Wnt pathway and mediate TCF1 methylation, which promotes THCA proliferation and migration.	Wang et al., 2020b
NPC	METTL3	Up	Via m6A modification, METTL3 can promote EZH2 expression and NPC progression. EZH2 can also inhibit CDKN1C and promote tumor cell proliferation.	Meng et al., 2020
	YTHDF1	Down	YTHDF1 inhibits BZLF1/BRLF1 to promote the recognition of EBV transcripts and inhibit NPC development.	Xia et al., 2021
HPSCC	YTHDF1	Up	YTHDF1 can promote the expression of TFRC, which promotes the entry of Fe3+ ions into cells and accelerates HPSCC formation.	Ye et al., 2020
OSCC	METTL3/IGF2BP1	Up	By recognizing m6A on the 3′ UTR of BMI-1, METTL3 can interact with IGF2BP1, which promotes BMI-1 expression and accelerates OSCC proliferation and metastasis.	Liu et al., 2020c
	ALKBH5	Up	DDX3 can increase ALKBH5 expression and promote FOXM1/NANOG via m6A modification, thereby inducing OSCC chemotherapy resistance.	Shriwas et al., 2020
0 Others	METTL3/14	Up	METTL3/14 can stabilize lncAROD, which promotes YBX1 and HNSCC.	Ban et al., 2020
	YTHDC2	Down	YTHDC2 can interact with several immune cell types in cancer and improve HNSCC prognosis.	Li et al., 2020
	METTL3	Down	METTL3-mediated deposition of m6A can accelerate SOCS recognition and promote IL2-STAT5 signaling pathway activation, which can maintain the immunosuppressive function of Treg and inhibit tumor progression.	Zhao and Cui, 2019

*THCA, thyroid carcinoma; NPC, nasopharyngeal carcinoma; HPSCC, hypopharynx squamous cell carcinoma; OSCC, oral squamous cell carcinoma.*

### Thyroid Carcinoma

Thyroid cancer accounts for 1% of all malignant tumors; however, its incidence varies greatly according to region, ethnicity, and sex. For instance, females are more prone to thyroid cancer than males (Cabanillas et al., 2016; Roman et al., 2017), while papillary carcinoma is the most common malignant thyroid tumor, especially among young adults, and is characterized by a low malignancy and good prognosis (Carling and Udelsman, 2014; Araque et al., 2017). Therefore, the precise molecular mechanisms underlying the occurrence and development of thyroid cancer must be elucidated to develop targeted therapies.

In Xu et al. (2020) identified various m6A-related differentially expressed genes (*METTL3*, *YTHDC2*, *HNRNPC*, *WTAP*, *YTHDF1*, *ALKBH5*, *METTL14*, *YTHDC1*, *FTO*, *ZC3H13*, *KIAA1429*, *YTHDF2*, and *RBM15*) between patients with thyroid cancer and normal patients from TCGA datasets. After validating the gene signature using three GEO datasets (GSE33630, GSE35570, and GSE60542), the authors concluded that m6A modification affects the prognosis of thyroid cancer. Moreover, Wang et al. (2020b) found that the expression of *METTL3*, *YTHDC1*, *FTO*, *METTL14*, *RBM15*, *YTHDF3*, *WTAP*, *HNRNPA2B1*, *ALKBH5*, *METTL16*, *YTHDC2*, *KAA1429*, *IGF2BP3*, *RBM15B*, and *YTHDF1* was significantly lower in thyroid cancer tissues than that in normal thyroid tissues using bioinformatics analysis, consistent with the findings of Hou et al. (2020). However, studies have also shown that METTL3 can induce the m6A mRNA modification of TCF1, a downstream effector of the classical Wnt pathway encoded by *TCF7*, by activating the Wnt pathway, thereby accelerating the progression of thyroid cancer (Wang et al., 2020c). Although these results indicate that METTL3 exerts contradictory effects on thyroid cancer, the precise underlying mechanism remains unclear. This may be due to the fact that thyroid carcinoma has multiple subtypes that present with different clinical and pathological features. Therefore, the role of m6A-modification related proteins in different thyroid cancer subtypes should be validated in future studies.

### Nasopharyngeal Carcinoma

Although chemotherapy and radiotherapy have improved the overall survival of patients with nasopharyngeal carcinoma, approximately 30% of treated patients develop metastasis or recurrence and their prognosis is often poor (Lee et al., 2017; Liu et al., 2018; Chen et al., 2019). Therefore, an improved understanding of the precise mechanisms underlying the occurrence and development of nasopharyngeal carcinoma is required to develop more effective treatment methods.

Epstein-Barr virus (EBV) is responsible for causing many types of malignant tumors and is widely associated with B-cell lymphoma, gastric cancer, and nasopharyngeal carcinoma (Shi et al., 2016; Young et al., 2016; Liu et al., 2019). Recently, the EBV transcriptome was studied in EBV-transformed lymphoblastoid and lymphoma cells, and the findings of this analysis revealed that m6A regulates EBV-associated tumorigenesis (Lang et al., 2019). Although the m6A modification was identified during the latent stage of EBV infection, the modification of EBV transcripts by the deposition of m6A was reduced during lytic infection. Increased EBNA3C levels have been shown to increase the expression and stability of METTL14, which can induce the proliferation and colony formation of EBV-positive cells (Zheng et al., 2021) and stabilize METTL3 via its interaction with RNAs (Klungland and Dahl, 2014; He and He, 2021). METTL3 is highly expressed in nasopharyngeal carcinoma tissues and affects the overall survival of patients with nasopharyngeal carcinoma. In addition, METTL3 can enhance the m6A modification of *EZH2* mRNA to increase the expression of EZH2 protein, an important component of the PRC2 complex that plays a role in gene silencing by depositing methyl groups on the lysine 27 residue of histone 3 (Zhong et al., 2013; Yamaguchi and Hung, 2014; Xu et al., 2019). Several studies have also shown that EZH2 can promote cell proliferation by inhibiting cyclin-dependent kinase (CDK) inhibitor 1C (CDKN1C) (Yang et al., 2009), which is inactivated by promoter DNA methylation in several human tumors (McGarvey et al., 2006; Fang et al., 2021), thereby promoting nasopharyngeal carcinoma development and increasing the malignancy of nasopharyngeal carcinoma cells (Meng et al., 2020).

In EBV-induced nasopharyngeal carcinoma, YTHDF1 has been shown to inhibit tumor development by downregulating BZLF1 and BRLF1, thereby reducing the stability of EBV transcripts, and interact with the RNA degradation complex to promote RNA degradation (Xia et al., 2021). The RNA degradation complex consists of two components, i.e., ZAP and DDX17. ZAP induces the degradation of target mRNAs, while DDX17 binds to ZAP-bound mRNAs to induce their degradation (Chen et al., 2008). However, a recent study demonstrated that the deletion of YTHDF2 and/or YTHDF3 can enhance the interaction between YTHDF1 and DDX17, suggesting that YTHDF1, YTHDF2, and YTHDF3 may compete for binding to DDX17 (Youn et al., 2018; Wu, 2020). These findings suggest that m6A-binding proteins have complex regulatory mechanisms in nasopharyngeal carcinoma; however, further experiments are required to determine their precise effects.

### Hypopharynx Squamous Cell Carcinoma (HPSCC)

HPSCC has the worst prognosis among all types of head and neck tumors as it is generally detected at a late stage due to a lack of potent biomarkers for early diagnosis (Gatta et al., 2015; Arends et al., 2020). The unique clinical and biological characteristics of HPSCC are attributed to its distinct anatomical location as well as genetic and transcriptome alterations (Hajek et al., 2017; Morris et al., 2017). For example, the co-occurrence of CCND1 and CDKN2A mutations and chromosomal instability markers have been associated with radiotherapy and chemotherapy outcomes in patients with advanced HPSCC (Yamashita et al., 2019). Although various genomic alterations have been reported to be associated with sensitivity to chemotherapy, targeted therapy, and ionizing radiation (Jiang et al., 2019), only a few specific biomarkers and therapeutic targets for HPSCC have been identified and validated. Therefore, a better understanding of the molecular mechanism underlying HPSCC progression is required to improve its treatment.

Several studies have reported the carcinogenic significance of transcription factor sets that regulate TFRC (Petronek et al., 2019), whose overexpression on the extracellular surface of the cell membrane of solid tumors results in increased iron uptake (Shen et al., 2018). Upon its release from the TF-TFRC complex into the cytoplasm, iron is reduced to its ferrous form by iron reductase *in vivo* (Ohgami et al., 2006; Gomes et al., 2012). YTHDF1 knockout was found to reduce TFRC protein expression in Detroit562 and FADU cells without affecting TFRC mRNA expression or protein stability, indicating that YTHDF1 upregulates TFRC translation via its methyltransferase domain. Moreover, high levels of YTHDF1 and TFRC have been associated with poor prognosis in patients receiving chemoradiotherapy or radiation (CCT/RT) adjuvant therapy and YTHDF1 has been shown to increase TFRC expression in HPSCC in an m6A-dependent manner (Ye et al., 2020). Although no other m6A modification-related molecules have yet been associated with HPSCC, m6A is thought to play an important role and future studies should be aimed at identifying the precise roles of various m6A-related proteins in HPSCC tumors.

### Oral Squamous Cell Carcinoma (OSCC)

OSCC is the most common type of oral and maxillofacial cancer. Although surgery and CCT/RT are associated with prolonged patient survival, the rate of overall survival remains low (Ferlay et al., 2010; Leemans et al., 2011; Hedberg et al., 2016). Previous studies have shown that ncRNA-mediated epigenetic modifications play an important role in OSCC via multiple signaling pathways (González-Ramírez et al., 2014; Jithesh et al., 2013; Bavle et al., 2016). For instance, the m6A writer protein METTL3 can recognize m6A residues on the 3’-UTR of BMI1 and bind to the m6A reader protein IGF2BP1 to promote BMI1 translation, thereby inducing OSCC proliferation and metastasis (Liu et al., 2020c). However, the functional mechanism of m6A in OSCC requires further study.

m6A also plays an important role in mediating the development of drug resistance in OSCC. The chemotherapy regimen most commonly used to treat OSCC is cisplatin, either alone or in combination with 5-fluorouracil and docetaxel (Lorch et al., 2011). However, resistance to chemotherapy can reduce its ability to treat OSCC, ultimately resulting in continued tumor growth (Wang et al., 2016c). DEAD-box helicase 3 X-linked (DDX3) is a human dead-box RNA helicase that is involved in RNA metabolism and translation (Tanner and Linder, 2001; Geissler et al., 2012). DDX3 directly regulates m6A through the methylase ALKBH5 and reduces the transcription of cancer stem cell transcription factor FOXM1 (Fork head box protein M1) and Nanog homeobox, resulting in chemotherapy drug resistance (Shriwas et al., 2020). Interestingly, the DDX3 inhibitor ketorolac can restore cisplatin-mediated cell death and significantly reduce tumor burden (Shriwas et al., 2020), although it remains unclear whether this mechanism of drug resistance reversal directly involves the m6A modification. Thus, further experimental evidence and clinical studies are warranted.

**FIGURE 3:**
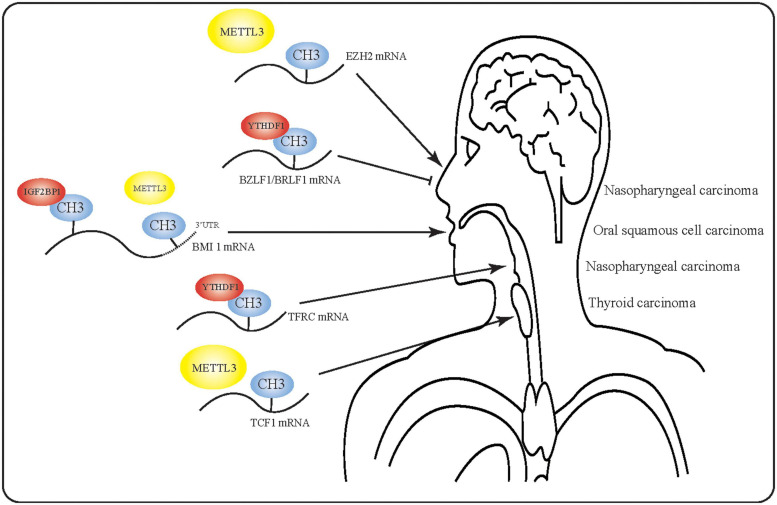
Mechanism of action of m6A-modified mRNA in head and neck squamous cell carcinoma. m6A-modified RNA plays multiple roles in various tumors. In nasopharyngeal carcinoma, m6A-modified RNA may even exert opposite effects due to the presence of multiple m6A targets.

Despite the fact that m6A has been implicated in the occurrence and progression of various cancers, m6A RNA modification has not been extensively studied in HNSCC. Indeed, there have been no reports of m6A modification in larynx, oropharynx, or nasal carcinoma. Furthermore, the precise mechanisms underlying m6A RNA modification in HNSCC are even less well understood. Although researchers have begun to study the effect of m6A in EBV, no studies have yet been conducted on the equally important Human Papilloma Virus (HPV). However, the findings discussed in this review suggest that m6A may play a key regulatory role in HNSCC (Figure 3) by promoting or inhibiting the occurrence and development of tumors, thus could help to predict tumor prognosis. Based on the discovered regulatory functions of non-coding RNA, we propose that future studies of m6A-modified RNA could help to elucidate the molecular events in HNSCC.

## Relationship Between m6A and Immune Cells in the Tumor Microenvironment

Several studies have demonstrated that tumor immune cells can perform vastly different or even opposite functions under the influence of the tumor microenvironment (Jiménez-Sánchez et al., 2017; Wagner et al., 2019; Luoma et al., 2020; Kolb et al., 2021), with some studies suggesting that m6A modification plays an important regulatory role in these immune cells. Here, we discuss the relationship between immune cell infiltration and m6A modification in HNSCC, with a view to providing directions for future studies.

Members of the SOCS gene family, which consists of *Cish*, *Socs1*, *Socs2, Socs3*, and *Asb2*, play important roles in the signal transduction of negative regulatory factors. Studies have shown that SOCS mRNAs can be m6A-modified and that these mRNAs are m6A targets in CD4^+^ T cells (Li et al., 2017c). In addition, the loss of METTL3 has been shown to reduce the overall levels of m6A-modified mRNA and cause the subsequent loss of specific SOCS gene transcription. Reduced m6A modification also enhances the stability of SOCS mRNA, increases the expression of SOCS proteins, and blocks cytokine signaling to inhibit tumor development and metastasis (Tong et al., 2018). Moreover, m6A can specifically regulate Tregs, which inhibit the tumor-killing function of CD8^+^T cells in the tumor microenvironment (Li and Rudensky, 2016). Therefore, the selective removal of m6A from tumor-infiltrating Tregs might increase the efficacy of tumor immunotherapies.

YTHDC2 expression is significantly correlated with B cells, CD4^+^T cells, neutrophils, and dendritic cells, but not with macrophage infiltration in HNSCC (Li et al., 2020), indicating that YTHDC2 may play an important role in immune cells in the tumor microenvironment, especially CD4^+^T cells and dendritic cells. Further analysis revealed that changes in the somatic copy number of recognized m6A regulator-based signals significantly affect the infiltration of B cells, CD4^+^T cells, CD8^+^T cells, neutrophils, macrophages, and dendritic cells in HNSCC (Yi et al., 2020b), consistent with the hypothesis that this m6A regulatory protein plays a key role in HNSCC development.

Prognostic survival curves obtained using the CIBERSORT-ABS and xCell algorithms showed that TFH or CD4^+^T cells with increased YTHDC2 expression and high immune infiltration were associated with a better HNSCC prognosis than those with reduced YTHDC2 expression and low immune infiltration. However, the survival curves obtained using EPIC and CIBERSORT algorithms indicated that patients with low YTHDC2 expression and low CD4^+^T or CD4^+^ natural T cell infiltration had a better prognosis than those with high YTHDC2 expression and high CD4^+^T or CD4^+^ natural T cell infiltration. Moreover, when the effect of YTHDC2 expression on HNSCC prognosis was evaluated, tumors with high YTHDC2 expression displayed a better prognosis than those with low YTHDC2 expression (Li et al., 2020). Together, these findings suggest that different CD4^+^T cell subsets may exert different effects on the prognosis of HNSCC.

## Potential Therapeutic Targets of m6A

Low levels of METTL3 or METTL14, key components of the RNA methyltransferase complex, have been shown to reduce the expression of m6A-modified *ADAM19* RNA, whereas high *ADAM19* RNA expression in glioblastoma stem cells causes glioblastoma (Cui et al., 2017). Thus, m6A-modified ADAM19 could represent a potential drug target for treating glioblastoma ass its downregulation could prevent tumor development. Huang et al. (2016) showed for the first time that m6A RNA methylation in circulating tumor cells is significantly higher than that in whole blood cells, which may help to elucidate the mechanism underlying cancer metastasis and provide novel insights into the diagnosis of early tumors. Interestingly, meclofenamic acid (MA) and FTO competitively bind to m6A-containing nucleotides and increase the expression of m6A-modified mRNA (Huang et al., 2015); therefore, it may be possible to develop a therapeutic target for cancer that indirectly regulates m6A levels via MA. It has also been reported that the miR-33a-directed targeting of METTL3 inhibits NSCLC cell proliferation; therefore, miR-33a could represent a potential therapeutic molecule for treating NSCLC (Du et al., 2017) and this interaction should be studied in more detail to develop novel therapeutic targets.

The direct causal relationship between m6A RNA methylation and its role in inhibiting or promoting tumor growth requires further study, since it remains unclear whether tumor progression can be altered via the regulation of m6A modification alone or in combination with other regulatory factors. Therefore, future studies focusing on m6A-related targets should not only be limited to the few identified enzymes affecting m6A modification, but also target genes and ncRNAs that are closely related to m6A.

## Discussion

This review of current literature revealed that the role of m6A modification varies greatly in different tumors. For example, m6A modification mainly affects RNA stability and protein expression at the post-transcriptional and translational levels in nasopharyngeal carcinoma, but affects post-translational protein modification in HPSCC rather than TFRC stability or mRNA expression. Interestingly, m6A modification plays a regulatory role in tumors via two main mechanisms: (1) the direct methylation of mRNA that encodes the regulated protein, which alters protein expression; and (2) the m6A modification of ncRNA, which affects tumor occurrence and development through ncRNA regulation. Although studies have shown that both of these mechanisms exist in HNSCC, the latter is seldom studied. At present, there are few studies on the regulatory mechanism involved in the m6A modifications in head and neck tumors. In the future, it is necessary to further clarify the key role of m6A in head and neck tumors, understand the mechanism of tumor occurrence and development comprehensively, and find more effective intervention measures and treatment approaches.

The role of m6A in various types of HNSCCs is being uncovered gradually and further studies could provide insights into this novel model of m6A-mediated epigenetic regulation and identify related diagnostic/therapeutic targets. Notably, the relationships between m6A modification, smoking, alcohol, and HPV, which are all closely related to head and neck tumors, require further study. In addition, the current bioinformatics results relating to thyroid cancer are inconsistent with those of molecular experiments, potentially due to a limited understanding of related molecular mechanisms and the broad classifications of thyroid cancer. Consequently, extensive studies are required to elucidate these molecular mechanisms and to develop more efficient methods for treating HNSCC.

## Author Contributions

F-YJ designed the study. F-YJ, L-MZ, and Y-JN collected data and aided in writing the manuscript. F-YJ, L-MZ, and X-JW edited the manuscript. Y-MZ provided direction and guidance through the preparation of this study and assisted in the revision of the manuscript. All authors read and approved the final manuscript.

## Conflict of Interest

The authors declare that the research was conducted in the absence of any commercial or financial relationships that could be construed as a potential conflict of interest.

## Abbreviations

AML: acute myeloid leukemia
ASF/SF2: alternative splicing factor/splicing factor 2
ALKBH5: alkB homolog 5
CCT/RT: chemoradiotherapy or radiation
CDKN1C: cyclin-dependent kinase inhibitor 1C
CDK: cyclin-dependent kinase
DDX3: DEAD-box helicase 3 X-linked
Dnmt3b: DNA methyltransferase 3 beta
eIF3: eukaryotic initiation factor 3
EBV: Epstein-Barr virus
FOXM1: fork head box protein M1
FTO: FTO alpha-ketoglutarate dependent dioxygenase
HPSCC: hypopharynx squamous cell carcinoma
HNRNP: heterogeneous nuclear ribonucleoprotein family protein
HNSCC: head and neck squamous cell carcinoma
ID4: inhibitor of DNA binding 4
m6A: N6-methyladenosine
MA: meclofenamic acid
NSUN2: NOP2/Sun RNA methyltransferase family member 2
OSCC: oral squamous cell carcinoma
RBM15: RNA binding motif protein 15
Sohlh2: spermatogenesis and oogenesis specific basic helix-loop-helix 2
SOCS: suppressor of cytokine signaling
YTH: YT521-B homolog
ZBTB16: zinc finger and BTB domain containing 16
ZFP217: zinc finger protein 217
3’-UTR: 3’-untranslated region.
